# Arterial Stiffness as a Predictor of Postoperative Intensive Care Unit (ICU) Mortality: An Observational Study

**DOI:** 10.7759/cureus.109812

**Published:** 2026-05-28

**Authors:** Panagiota Gatsori, George Intas, Theodoros A Katsoulas, Eleni Theodosopoulou, Dimitrios Cholevas, Ilias Skopelitis, Pantelis I Stergiannis

**Affiliations:** 1 Intensive Care Unit, General Hospital of Nikaia, Athens, GRC; 2 Department of Nursing, General Hospital of Nikaia, Athens, GRC; 3 Faculty of Nursing, National and Kapodistrian University of Athens, Athens, GRC; 4 Intermediate Care Unit, KAT General Hospital of Attica, Athens, GRC; 5 Department of Pathology, General Hospital of Nikaia, Athens, GRC

**Keywords:** aix@75, arterial stiffness, augmentation index, hemodynamics, mortality, postoperative icu, pulse pressure

## Abstract

Background

Arterial stiffness has emerged as a key determinant of cardiovascular risk, yet its prognostic impact in postoperative intensive care unit (ICU) patients remains unclear. This study evaluated the association of arterial stiffness indices (augmentation index (AIX@75), pulse pressure (PP)) with ICU mortality in postoperative surgical patients.

Methods

This was a prospective observational study including 117 postoperative ICU patients. Demographics, comorbidities, Acute Physiology and Chronic Health Evaluation II (APACHE II), Sequential Organ Failure Assessment (SOFA), laboratory values, hemodynamics, central pressures, AIX, AIX@75, and PP were recorded. Mortality was the primary endpoint. Statistical analyses included Mann-Whitney U tests, receiver operating characteristic (ROC) curve analysis, logistic regression, and Kaplan-Meier survival curves.

Results

Overall mortality was 38.5% (45/117). Non-survivors had significantly higher APACHE II scores (p = 0.001) and PP (p = 0.005). ROC curves identified AIX@75 as a strong mortality predictor (area under the curve (AUC) = 0.78), with an optimal cut-off of ≥23.5 (sensitivity = 82%; specificity = 68%). PP had modest discriminatory ability (AUC = 0.62) with a cut-off of ≥42.5 (sensitivity = 84.4%; specificity = 36.1%). Logistic regression showed AIX@75 significantly predicted mortality (OR = 1.037 per unit; p = 0.006), while PP showed a borderline association (p = 0.062). Kaplan-Meier curves demonstrated significantly reduced survival in high-AIX@75 patients (p < 0.001) and a trend for PP (p = 0.069).

Conclusions

AIX@75 is a robust independent predictor of postoperative mortality in ICU patients, outperforming PP. Arterial stiffness assessment may provide valuable early risk stratification in postoperative critical care.

## Introduction

Postoperative patients admitted to the intensive care unit (ICU) experience substantial hemodynamic stress, systemic inflammation, and risk of early organ dysfunction. Mortality in this population remains significant, highlighting the need for early and precise risk stratification. Conventional severity scoring systems such as Acute Physiology and Chronic Health Evaluation II (APACHE II) and Sequential Organ Failure Assessment (SOFA) reflect global physiological status but do not incorporate vascular pathophysiology. Consequently, they may fail to detect early circulatory impairment arising from altered arterial compliance and wave reflection patterns, factors increasingly recognized as central to the evolution of postoperative critical illness.

Arterial stiffness reflects cumulative structural and functional changes in the arterial wall, including elastin fragmentation, collagen deposition, medial calcification, and endothelial dysfunction. These processes accelerate pulse wave velocity (PWV), increase central systolic pressure, and augment pulse pressure (PP), raising left ventricular afterload and reducing diastolic coronary perfusion [1-3]. As these alterations impair organ perfusion and cardiac efficiency, arterial stiffness has been widely recognized as a major determinant of cardiovascular morbidity and mortality [4-6].

A substantial body of literature has demonstrated independent associations between increased arterial stiffness, usually assessed by PWV, and all-cause mortality in populations such as hypertensive patients, diabetics, individuals with chronic kidney disease, and the elderly [7-11]. These associations reflect the role of arterial stiffness as a surrogate marker of vascular aging and cumulative cardiovascular risk burden.

Pulse wave analysis (PWA) enables the non-invasive assessment of central hemodynamics by applying generalized transfer functions to radial tonometric waveforms [12]. Derived metrics such as central systolic and diastolic pressure, central PP, augmentation pressure, and augmentation index (AIX) reflect the interaction between forward and reflected waves and provide insight into arterial stiffness and ventricular-vascular coupling [13,14]. Central hemodynamics are more closely associated with cardiovascular outcomes and organ dysfunction than peripheral pressures [15,16]. AIX, especially when standardized to heart rate (AIX@75), captures reflective wave burden and has demonstrated strong prognostic value across diverse populations [17,18].

In critically ill and postoperative patients, vascular tone and elasticity may be acutely altered by systemic inflammation, anesthesia, surgical trauma, fluid shifts, and vasopressor therapy [19-21]. Recent studies in sepsis, acute kidney injury, cardiac surgery, and severe infections have shown that increased arterial stiffness is associated with multiorgan failure and short-term mortality [21,22]. These findings suggest that central arterial stiffness may reflect acute circulatory stress during critical illness and could serve as a meaningful prognostic marker.

Despite extensive research in other populations, the prognostic value of arterial stiffness in postoperative ICU patients remains largely unexplored. The postoperative period is characterized by dynamic fluctuations in vascular tone and inflammation, which may unmask underlying vascular vulnerability. Because PWA is non-invasive and rapidly obtained at the bedside, arterial stiffness assessment may offer valuable prognostic information in this setting.

The primary objective of this study was to evaluate whether central arterial stiffness indices, specifically AIX standardized to a heart rate of 75 bpm (AIX@75) and PP, are associated with ICU mortality in postoperative surgical patients admitted to intensive care. Secondary objectives were to investigate the relationship between arterial stiffness indices and established severity markers, including APACHE II and SOFA scores, to identify demographic, clinical, and perioperative factors associated with increased arterial stiffness in postoperative ICU patients. Finally, the study attempted to assess the prognostic performance of arterial stiffness parameters using receiver operating characteristic (ROC) curve, survival, and regression analyses.

## Materials and methods

Study design and ethics

This prospective observational cohort study was conducted over a 12-month period in the ICU of the General Hospital of Nikaia-Piraeus "Agios Panteleimon", Athens, Greece. Ethical approval was obtained from the Scientific Council of the General Hospital of Nikaia-Piraeus (approval number: 38887/25-8-23). The study was conducted in accordance with the Declaration of Helsinki. Written informed consent was obtained from all participants or their next of kin. Confidentiality and anonymity were strictly maintained, and data were used for research purposes in accordance with ethical guidelines.

Study population

Patients were considered eligible for inclusion if they were ≥18 years of age and were admitted to the ICU following either elective or emergency surgical procedures. Eligibility further requires the ability of patients to undergo PWA within the first 24 hours of ICU admission. Exclusion criteria included atrial fibrillation, significant arrhythmias, severe aortic regurgitation, hemodynamic instability precluding reliable PWA measurement, and poor tonometric signal quality despite repeated attempts.

Data collection

Demographics, comorbidities, surgical type, hemodynamics, and laboratory values were recorded. APACHE II and SOFA scores were calculated at admission (SOFA1) and at 48 hours (SOFA2).

PWA

PWA was performed within the first 24 hours following ICU admission and after the initial hemodynamic stabilization. Measurements were obtained with patients in the supine position after at least 10 minutes of rest. Radial artery applanation tonometry was performed using the SphygmoCor system (AtCor Medical, Sydney, Australia). Calibration was performed using simultaneously recorded brachial systolic and diastolic blood pressures obtained with an automated oscillometric device. Radial artery waveforms transformed into central aortic waveforms via validated transfer functions [11].

Three consecutive recordings were obtained for each participant, and the average value was used for analysis. Only recordings with an operator index of ≥80% were considered acceptable. Patients receiving vasoactive agents at the time of measurement were not excluded; however, vasoactive medication use and hemodynamic support were recorded as part of routine ICU monitoring.

Recorded parameters included the following: AIX and AIX@75, central systolic/diastolic pressure, and PP.

Patients with incomplete arterial stiffness measurements or inadequate waveform quality despite repeated attempts were excluded from the final analysis. No imputation methods were applied for missing data due to the observational nature of the study.

Outcomes

The primary outcome was ICU mortality. ICU length of stay was used as the survival time for Kaplan-Meier analysis.

Statistical methods

Statistical analysis was performed using IBM SPSS Statistics for Windows, V. 26.0 (IBM Corp., Armonk, NY, USA). Continuous variables were assessed for normality using the Shapiro-Wilk test. Because most variables demonstrated non-normal distribution, data are presented as mean ± standard deviation (SD) or median (interquartile range (IQR)), as appropriate.

Continuous variables were compared using the Mann-Whitney U test, whereas categorical variables were analyzed using the chi-squared test or Fisher's exact test. ROC curve analysis was used to evaluate the discriminative performance of arterial stiffness indices for ICU mortality. Optimal cut-off values were determined using the Youden index.

Survival analysis was performed using Kaplan-Meier curves, with ICU length of stay used as the time-to-event variable and mortality as the event of interest. Survival curves were compared using the log-rank test.

Variables with p < 0.10 in univariable analysis and variables considered clinically relevant were entered into the multivariable logistic regression model. Odds ratios (ORs) and hazard ratios (HRs) are presented with corresponding 95% confidence intervals (CIs). Statistical significance was defined as p < 0.05.

## Results

Study population

A total of 117 postoperative ICU patients were enrolled. Baseline characteristics are presented in Table 1.

**Table 1:** Baseline characteristics of the study population Data are presented as mean ± SD, median (IQR), or number (n, %), as appropriate. APACHE II: Acute Physiology and Chronic Health Evaluation II; SOFA: Sequential Organ Failure Assessment; CRP: C-reactive protein; HS-TnI: high-sensitivity troponin I; SD: standard deviation; IQR: interquartile range; AIX: augmentation index; ICU: intensive care unit

Variable	Mean ± SD, median (IQR), or number (n, %)
Age (years)	60.83 ± 17.66
Gender	Male: 79 (67.5%); female: 38 (32.5%)
BMI (kg/m²)	27.59 ± 7.00
APACHE II score	21.64 ± 7.15
SOFA score at admission (SOFA1)	7.62 ± 2.99
SOFA score at 48 hours (SOFA2)	7.50 ± 3.20
Albumin (g/dL)	2.66 ± 0.62
CRP (mg/L)	121.9 ± 208.8
HS-TnI (ng/L)	347.3 ± 1117.1
Pulse pressure (mmHg)	53.54 ± 15.53
AIX (%)	133.0 ± 28.3
AIX@75 (%)	21.73 ± 14.49
ICU length of stay (days)	21 (IQR 10-44)

Mortality was 38.5% (45/117). Significant differences were observed between survivors and non-survivors (Tables 2-4). 

**Table 2:** Comparison of continuous demographic and clinical variables between survivors and non-survivors Data are presented as mean ± SD. Group comparisons were performed using the Mann-Whitney U test. A p-value of <0.05 was considered statistically significant. APACHE II: Acute Physiology and Chronic Health Evaluation II; SOFA: Sequential Organ Failure Assessment; CRP: C-reactive protein; HS-TnI: high-sensitivity troponin I; SD: standard deviation

Variable	Survivors (n = 72)	Non-survivors (n = 45)	P-value
Age (years)	56.57 ± 17.53	67.64 ± 15.79	<0.001
BMI (kg/m²)	27.40 ± 7.13	27.90 ± 6.87	0.549
APACHE II score	19.60 ± 6.80	24.91 ± 6.49	0.001
SOFA score at admission (SOFA1)	7.11 ± 2.84	8.44 ± 3.06	0.034
SOFA score at 48 hours (SOFA2)	6.90 ± 3.05	8.44 ± 3.23	0.009
Albumin (g/dL)	2.77 ± 0.60	2.48 ± 0.62	0.022
CRP (mg/L)	100.27 ± 108.39	156.55 ± 306.60	0.434
HS-TnI (ng/L)	238.08 ± 455.17	522.14 ± 1704.36	0.396

**Table 3:** Comparison of hemodynamic and arterial stiffness parameters between survivors and non-survivors Data are presented as mean ± SD. Group comparisons were performed using the Mann-Whitney U test. A p-value of <0.05 was considered statistically significant. SBP: systolic blood pressure; AIX: augmentation index; SD: standard deviation

Variable	Survivors (n = 72)	Non-survivors (n = 45)	P-value
Pulse pressure (mmHg)	50.13 ± 14.88	59.00 ± 15.12	0.005
Central SBP (mmHg)	119.32 ± 17.48	126.76 ± 18.62	0.048
AIX (%)	129.00 ± 24.41	139.47 ± 32.86	0.007
AIX@75 (%)	18.25 ± 13.95	27.29 ± 13.72	<0.001

**Table 4:** Comparison of categorical variables between survivors and non-survivors Data are presented as numbers (n, %). Group comparisons were performed using the chi-squared test or Fisher's exact test, as appropriate. High arterial stiffness was defined as AIX@75 ≥23.5. A p-value of <0.05 was considered statistically significant. AIX: augmentation index

Variable	Survivors, n (%)	Non-survivors, n (%)	P-value
Male sex	50 (69.4%)	29 (64.4%)	0.720
Elective surgery	47 (65.3%)	15 (33.3%)	0.001
Hypertension	26 (36.1%)	29 (64.4%)	0.005
Diabetes mellitus	16 (22.2%)	22 (48.9%)	0.005
Smoking	28 (38.9%)	20 (44.4%)	0.431
Alcohol use	11 (15.3%)	7 (15.6%)	0.761
Hypercholesterolemia	52 (72.2%)	30 (66.7%)	0.666
Organ failure	26 (36.1%)	20 (44.4%)	0.482
High arterial stiffness	30 (41.7%)	35 (77.8%)	<0.001

Non-survivors were significantly older compared with survivors (67.64 ± 15.79 vs 56.57 ± 17.53 years; U = 1015.0; p < 0.001). Disease severity scores were also significantly higher in non-survivors, including APACHE II (24.91 ± 6.49 vs 19.60 ± 6.80; U = 1015.0; p = 0.001), SOFA score at admission (SOFA1) (8.44 ± 3.06 vs 7.11 ± 2.84; p = 0.034), and SOFA score at 48 hours (SOFA2) (8.44 ± 3.23 vs 6.90 ± 3.05; p = 0.009).

Albumin levels were significantly lower in non-survivors (2.48 ± 0.62 vs 2.77 ± 0.60 g/dL; p = 0.022). 

Regarding arterial stiffness parameters, non-survivors demonstrated significantly higher PP (59.00 ± 15.12 vs 50.13 ± 14.88 mmHg; U ≈ 1200; p = 0.005), central systolic blood pressure (126.76 ± 18.62 vs 119.32 ± 17.48 mmHg; p = 0.048), AIX (139.47 ± 32.86 vs 129.00 ± 24.41; p = 0.007), and AIX@75 (27.29 ± 13.72 vs 18.25 ± 13.95; p < 0.001). Categorical analysis showed that emergency surgery (χ² = 10.8; p = 0.001), hypertension (χ² ≈ 7.8; p = 0.005), diabetes mellitus (χ² ≈ 7.9; p = 0.005), and increased arterial stiffness (χ² ≈ 18.5; p < 0.001) were significantly more frequent in non-survivors.

Arterial stiffness analysis

Arterial stiffness indices demonstrated a significant association with ICU mortality, with AIX@75 emerging as the strongest predictor among the evaluated hemodynamic parameters.

ROC curve analysis (Table 5) demonstrated that AIX@75 had good discriminative ability for ICU mortality prediction, with an area under the curve (AUC) of 0.719 (95% CI: 0.616-0.822; p < 0.001). The optimal cut-off value was ≥23.5, yielding a sensitivity of 73.3% and a specificity of 66.7%.

**Table 5:** ROC curve analysis for the prediction of ICU mortality according to arterial stiffness indices ROC curve analysis evaluating the discriminative performance of arterial stiffness indices for ICU mortality prediction. Results are presented as AUC with corresponding 95% CI. Optimal cut-off values were determined using ROC curve analysis. A p-value of <0.05 was considered statistically significant. ICU: intensive care unit; ROC: receiver operating characteristic; AUC: area under the curve; CI: confidence intervals; AIX: augmentation index; PP: pulse pressure

Variable	AUC (95% CI)	SE	P-value	Cut-off	Sensitivity (%)	Specificity (%)
AIX@75 (%)	0.719 (0.616-0.822)	0.052	<0.001	≥23.5	73.3	66.7
PP (mmHg)	0.656 (0.555-0.758)	0.052	0.005	≥42.5	84.4	36.1

PP also demonstrated statistically significant discriminatory ability, although its predictive performance was inferior to that of AIX@75. The AUC for PP was 0.656 (95% CI: 0.555-0.758; p = 0.005), with an optimal cut-off value of ≥42.5, corresponding to a sensitivity of 84.4% and a specificity of 36.1%.

Kaplan-Meier survival analysis (Table 6) further demonstrated the prognostic significance of arterial stiffness indices. Patients with elevated AIX@75 (≥23.5) exhibited markedly reduced survival compared with those with lower values, with median ICU survival decreasing from 110 days to 36 days. This difference was highly statistically significant (log-rank χ² = 16.785; p < 0.001).

**Table 6:** Kaplan-Meier survival analysis according to arterial stiffness indices Kaplan-Meier survival analysis evaluating ICU survival according to arterial stiffness categories. ICU mortality was considered the event of interest, and ICU length of stay was used as the time-to-event variable. Survival differences between groups were evaluated using the log-rank test. A p-value of <0.05 was considered statistically significant. AIX: augmentation index; PP: pulse pressure; ICU: intensive care unit

Variable	Groups	Median survival (days)	Log-rank χ²	P-value
AIX@75 (%)	<23.5 vs ≥23.5	110 vs 36	16.785	<0.001
PP (mmHg)	<42.5 vs ≥42.5	110 vs 67	≈3.3	0.069

In contrast, PP-based stratification demonstrated only a non-significant trend toward reduced survival, with median survival decreasing from 110 days to 67 days (log-rank χ² ≈ 3.3; p = 0.069). Although PP showed moderate discriminatory ability in ROC curve analysis, its prognostic performance in time-to-event survival analysis appeared inferior to that of AIX@75.

Univariable logistic regression analysis (Table 7) demonstrated that AIX@75 was significantly associated with ICU mortality. Specifically, each one-unit increase in AIX@75 was associated with a 3.7% increase in the odds of death (OR = 1.037; 95% CI: 1.011-1.064; p = 0.006).

**Table 7:** Univariable logistic regression analysis for ICU mortality Univariable logistic regression analysis evaluating the association between arterial stiffness parameters and ICU mortality. Results are presented as OR with corresponding 95% CI. A p-value of <0.05 was considered statistically significant. AIX: augmentation index; PP: pulse pressure; ICU: intensive care unit; OR: odds ratios; CI: confidence intervals

Variable	OR (95% CI)	P-value
AIX@75 (%)	1.037 (1.011-1.064)	0.006
PP (mmHg)	1.018 (0.999-1.038)	0.062

In contrast, PP demonstrated only borderline statistical significance (OR = 1.018; 95% CI: 0.999-1.038; p = 0.062), suggesting a weaker association with ICU mortality compared with AIX@75.

Multivariable logistic regression analysis (Table 8) demonstrated that APACHE II score and elective surgery remained independently associated with ICU mortality. Elective surgery was associated with significantly lower odds of ICU mortality (adjusted OR = 0.161; 95% CI: 0.060-0.429; p < 0.001), while higher APACHE II scores independently increased mortality risk (adjusted OR = 1.097; 95% CI: 1.019-1.181; p = 0.014).

**Table 8:** Multivariable logistic regression analysis for the predictors of ICU mortality Multivariable logistic regression analysis evaluating the independent predictors of ICU mortality after adjustment for clinically relevant covariates. Variables with clinical relevance and/or statistical significance in univariable analysis were included in the multivariable model. Results are presented as adjusted OR with corresponding 95% CI. A p-value of <0.05 was considered statistically significant. AIX: augmentation index; PP: pulse pressure; ICU: intensive care unit; OR: odds ratios; CI: confidence intervals; APACHE II: Acute Physiology and Chronic Health Evaluation II

Variable	Univariate p-value	Adjusted OR	95% CI	P-value
AIX@75 (%)	0.001	1.040	0.999-1.081	0.055
PP (mmHg)	0.003	1.020	0.984-1.058	0.273
Age (years)	0.001	1.026	0.993-1.060	0.128
APACHE II score	<0.001	1.097	1.019-1.181	0.014
Elective surgery	0.001	0.161	0.060-0.429	<0.001

AIX@75 demonstrated a borderline independent association with ICU mortality after adjustment for age, APACHE II score, PP, and surgical type (adjusted OR = 1.040; 95% CI: 0.999-1.081; p = 0.055). PP and age did not remain statistically significant in the multivariable model.

Univariable Cox proportional hazards regression analysis (Table 9) demonstrated that AIX@75 was significantly associated with time to ICU mortality. Specifically, each one-unit increase in AIX@75 was associated with a 3.7% increase in the hazard of death (HR = 1.037; 95% CI: 1.011-1.064; p = 0.006).

**Table 9:** Univariable Cox proportional hazards regression analysis for ICU mortality Univariable Cox proportional hazards regression analysis evaluating the association between arterial stiffness indices and time to ICU mortality. Results are presented as regression coefficients (B), standard error (SE), hazard ratios (HR = Exp(B)), corresponding 95% CI, Wald statistics, and p-values. ICU length of stay was used as the time-to-event variable, and ICU mortality was considered the event of interest. A p-value of <0.05 was considered statistically significant. AIX: augmentation index; PP: pulse pressure; ICU: intensive care unit; CI: confidence intervals

Variable	B	SE	Wald	HR = Exp(B)	95% CI for HR	P-value	Omnibus χ²
AIX@75 (%)	0.036	0.013	7.610	1.037	1.011-1.064	0.006	8.559
PP (mmHg)	0.018	0.010	3.479	1.018	0.999-1.038	0.062	3.440

Given that AIX@75 is a continuous physiological variable, even modest elevations translated into clinically meaningful increases in mortality risk. When expressed per 10-unit increase, this corresponded to an approximate 44% increase in mortality risk (HR¹⁰ ≈ 1.44).

In contrast, PP demonstrated only borderline statistical significance (HR = 1.018; 95% CI: 0.999-1.038; p = 0.062), indicating weaker prognostic performance compared with AIX@75.

Importantly, these findings are consistent with both the ROC curve and Kaplan-Meier analyses, further supporting the prognostic relevance of AIX@75 in postoperative ICU patients. While PP may capture aspects of global hemodynamic burden, AIX@75 appears to more accurately reflect the complex interaction between ventricular function, arterial wave reflections, and vascular dysfunction.

Taken together, the Cox regression findings reinforce the concept that central arterial stiffness represents an important pathophysiological determinant of adverse outcomes and may provide clinically meaningful prognostic information beyond conventional hemodynamic indices.

## Discussion

The present study investigated the prognostic significance of arterial stiffness indices in postoperative ICU patients and demonstrated that central arterial stiffness, as assessed by AIX@75, was significantly associated with ICU mortality. Across multiple analytical approaches, including ROC curve analysis, survival analysis, logistic regression, and Cox proportional hazards modeling, AIX@75 consistently demonstrated superior prognostic performance compared with PP, highlighting the importance of central hemodynamic assessment in critically ill surgical populations.

One of the most notable findings of this study is the superior discriminative ability of AIX@75 compared with PP. ROC curve analysis demonstrated that AIX@75 achieved a higher AUC, indicating better capacity to distinguish between survivors and non-survivors. Although PP is widely used as a surrogate marker of arterial stiffness, its relatively lower discriminative performance in this study suggests that peripheral measures of pulsatile load may not adequately capture the complexity of vascular dysfunction in critically ill patients. In contrast, AIX@75, as an index of wave reflection and ventricular-vascular interaction, appears to provide a more integrative representation of arterial function.

The survival analysis further reinforced these findings. Patients with elevated AIX@75 exhibited markedly reduced survival, with a substantial decrease in median ICU survival time. The clear separation of Kaplan-Meier curves and the highly significant log-rank test underscore the strong association between increased arterial stiffness and adverse outcomes. In contrast, PP-based stratification demonstrated only a non-significant trend toward reduced survival, further emphasizing its limited prognostic utility in time-to-event analysis. These findings suggest that central arterial stiffness is not merely a marker of chronic vascular aging but may also reflect acute pathophysiological processes influencing survival during critical illness.

Importantly, the results of the multivariable logistic regression analysis demonstrated that AIX@75 showed a borderline independent association with ICU mortality after adjustment for key clinical confounders, including age, disease severity (APACHE II), PP, and surgical type. Although statistical significance was attenuated after adjustment, the observed association suggests that arterial stiffness may capture a dimension of physiological vulnerability not fully reflected by conventional severity scoring systems. While APACHE II remained an independent predictor of ICU mortality, the persistent association between AIX@75 and adverse outcomes supports the potential relevance of vascular dysfunction in determining prognosis among critically ill postoperative patients.

The Cox proportional hazards analysis further strengthened these observations by demonstrating that AIX@75 was significantly associated with time-dependent mortality risk. The observed increase in hazard per unit rise in AIX@75 indicates that arterial stiffness may influence not only whether death occurs but also when it is likely to occur during the ICU stay. This temporal dimension of risk is particularly important in critical care, where early identification of high-risk patients may guide monitoring intensity and therapeutic interventions.

In contrast, PP demonstrated only borderline significance across both logistic and Cox regression analyses. Although PP reflects arterial compliance, it is influenced by multiple dynamic factors in the ICU setting, including fluid resuscitation, vasoactive medication use, and rapid changes in vascular tone. These factors may attenuate its ability to serve as a stable prognostic marker. The discrepancy between AIX@75 and PP observed in this study supports the notion that central hemodynamic indices may provide more reliable and clinically relevant information than peripheral pressure measurements.

From a pathophysiological perspective, increased arterial stiffness leads to earlier wave reflection, augmentation of central systolic pressure, and increased left ventricular afterload while simultaneously reducing diastolic coronary perfusion. In the postoperative setting, where patients are already exposed to systemic inflammation, hemodynamic instability, and metabolic stress, these alterations may exacerbate organ hypoperfusion and contribute to the development of multiorgan dysfunction. Thus, AIX@75 may reflect both pre-existing vascular damage and acute circulatory impairment, explaining its association with mortality.

The clinical implications of these findings are substantial. AIX@75 is a non-invasive, rapidly obtainable parameter that can be assessed at the bedside using PWA. Its integration into early postoperative assessment may improve risk stratification and allow clinicians to identify patients at increased risk of deterioration. Moreover, arterial stiffness could represent a potential therapeutic target, although further research is required to determine whether interventions aimed at improving vascular function can translate into improved clinical outcomes.

Despite these strengths, several limitations should be acknowledged. First, the relatively small sample size and single-center design may limit the generalizability of the findings. Second, arterial stiffness measurements were obtained at a single postoperative time point and therefore do not reflect dynamic vascular changes during the ICU course. Third, vasoactive medications, fluid administration, and perioperative hemodynamic interventions may have influenced arterial stiffness indices and could not be fully standardized. Additionally, although multivariable adjustment was performed, residual confounding cannot be excluded. Finally, the observational design precludes conclusions regarding causality between arterial stiffness and mortality outcomes.

In conclusion, this study demonstrates that central arterial stiffness, as measured by AIX@75, is significantly associated with ICU mortality in postoperative patients. Compared with PP, AIX@75 demonstrated superior prognostic performance and appeared to capture clinically relevant aspects of cardiovascular dysfunction not fully reflected in conventional indices. These findings support the potential role of arterial stiffness assessment in postoperative critical care and highlight the need for further large-scale studies to validate its prognostic utility and clinical applicability.

## Conclusions

This study demonstrates that central arterial stiffness, as assessed by AIX@75, is significantly associated with ICU mortality in postoperative patients. Across multiple analytical approaches, including ROC curve analysis, survival analysis, logistic regression, and Cox proportional hazards modeling, AIX@75 consistently demonstrated superior prognostic performance compared with PP.

The findings indicate that arterial stiffness may capture important aspects of cardiovascular dysfunction that are not adequately reflected by conventional hemodynamic parameters or established severity scores. In contrast, PP exhibited weaker and less consistent predictive performance, underscoring the potential importance of central rather than peripheral indices of vascular function in the critical care setting.

The incorporation of arterial stiffness assessment into early postoperative evaluation may enhance risk stratification and facilitate the identification of high-risk patients. Given its non-invasive and bedside applicability, AIX@75 may represent a promising tool for improving clinical decision-making in ICU practice.

Further large-scale, multicenter studies are warranted to validate these findings and to determine whether targeted interventions aimed at improving arterial function can translate into better clinical outcomes.
